# Identifying the Toxidrome of Ivermectin Toxicity

**DOI:** 10.7759/cureus.42603

**Published:** 2023-07-28

**Authors:** Jon Stewart H Dy, Dan N Juangco

**Affiliations:** 1 Institute for Neurosciences, St. Luke's Medical Center, Quezon City, PHL

**Keywords:** activated charcoal, prophylaxis and treatment, toxicity, ivermectin, covid-19

## Abstract

Ivermectin is an antiparasitic drug that has been used as an alternative for prophylaxis and treatment of COVID-19 infection. The adverse effects from supratherapeutic doses of ivermectin can include non-neurological and neurological symptoms. In this study, we report the case of a 52-year-old Filipino male with newly diagnosed diabetes mellitus who developed a subacute history of fever, cough, and generalized weakness, causing him to self-medicate with supratherapeutic doses of ivermectin and thereafter subsequently developed a decrease in sensorium, restlessness, and complex visual hallucinations. Significant laboratory examinations showed hyperglycemia, mild hyponatremia, positive SARS-CoV2 reverse transcriptase polymerase chain reaction test, and bilateral pneumonia on chest radiograph. He was subsequently started on antibiotics, a high-flow nasal cannula, and given two doses of activated charcoal. During the first 24 hours of hospital admission, there was a significant improvement in the patient's sensorium with a resolution of restlessness and visual hallucinations. During the rest of the hospitalization, his respiratory symptoms improved, and he was subsequently discharged. Clinical outcome in our patient after administration of activated charcoal and completion of antibiotics showed an overall improvement in symptoms and without any neurologic sequelae.

## Introduction

Ivermectin is a derivative of avermectins and an antiparasitic drug that acts mainly by binding to glutamate-gated chloride channels, causing an increase in membrane permeability that leads to neuromuscular paralysis and parasite death [1]. Reports from in vitro studies have suggested that ivermectin can interfere with the attachment of SARS-CoV-2 spike protein to the human cell membrane, inhibition of host transport proteins that the virus uses to evade the antiviral response, and some studies suggest anti-inflammatory properties [2]. Despite this proposed clinical benefit, its use is limited to several tropical diseases such as scabies, helminthiases, and onchocerciasis. It is not approved by the Food and Drug Administration (FDA) for the treatment of any viral infection [1,2]. At present, there is insufficient evidence to recommend or discourage the use of ivermectin for the prevention and treatment of COVID-19 infection. Although the drug is generally well tolerated, non-neurological adverse effects may include nausea, vomiting, diarrhea, pruritus, and dizziness, while neurological adverse effects may include headache, encephalopathy, confusion, stupor, coma, dizziness, vertigo, and tremors [3, 4]. There are no available data on the therapeutic window of ivermectin. A case report shows that those with human ATP-binding cassette subfamily B member 1 (ABCB1) transporter nonsense mutations are those at risk for developing toxicity even with the intake of therapeutic doses of ivermectin [4]. Although studies on the neurological adverse effects of ivermectin are not locally available in the Philippines, a pharmacovigilance case series study showed serious adverse reactions such as toxidermias, encephalopathies, and confusional disorders after Ivermectin exposure [3]. In this study, we report the first locally-documented case of ivermectin overdose after intake of more than 10x the recommended maximum therapeutic dose of ivermectin and attempt to elucidate the possible adverse drug reactions from ivermectin toxicity.

## Case presentation

This patient is E.R., a 52-year-old male from the Philippines with a significant medical history of newly-diagnosed diabetes mellitus (glycohemoglobin level 13.2%). He presented with a decrease in sensorium. He works as a dentist and is independent in all activities of daily living. He is not on any maintenance medications, and he has no other known comorbid illnesses. He has poor health-seeking behavior, and he completed his primary doses of AstraZeneca COVID-19 vaccination 1.5 months prior to hospital admission at St. Luke's Medical Center. He had no other known vices, such as smoking and alcoholism.

On August 13, 2021, he started experiencing high-grade fever, nonproductive cough, generalized body weakness, and a decrease in appetite, for which febrile episodes would resolve with as-needed antipyretics. Interim history showed persistence of symptoms. On August 26, 2021, because of the persistence of symptoms, he self-medicated with 108 mg of ivermectin (12mg per tablet, a total of nine doses given) with no noted improvement of symptoms. On August 27, 2021, his symptoms had further worsened; he again self-medicated with 216 mg of ivermectin (12mg per tablet, a total of 18 doses given, cumulative dose of 4mg/kg taken in a 24-hour time period), and approximately five hours after intake, he then became drowsy, restless, agitated, confused and had experienced complex visual hallucinations, tremulousness, and gait instability. These symptoms eventually prompted hospital admission. On assessment, his systemic physical examination was significant for high oxygen requirement (on high flow nasal cannula with flow rate 30 liters per minute, FiO2 requirement of 100%), stable other vital signs, no signs of cardiorespiratory distress, a clinically hypovolemic state, and coarse bilateral crackles. The rest of his systemic physical examination was unremarkable. There were no noted skin or oral lesions, rashes, pallor, jaundice, or organomegaly. Neurological examination showed nonsustained eye opening to vigorous stimulation, inconsistent regard, unable to follow commands with psychomotor agitation, pupils 3 mm isochoric and briskly reactive to light, midline gaze, roving eye movements, no facial asymmetry, limited verbal output with no noted involuntary movements, with spontaneous and symmetric movement of all extremities. He was able to localize to pain on the bilateral upper extremities and withdraw to pain on the bilateral lower extremities, with supple neck, and no pathologic reflexes, while full cerebellar and gait examinations were difficult to assess given the level of consciousness. The differential diagnosis on admission included toxic-metabolic encephalopathy versus COVID-related septic and hypoxic encephalopathy, to rule out a central nervous system (CNS) infection (viral encephalitis) and nonconvulsive seizures.

**Figure 1:**
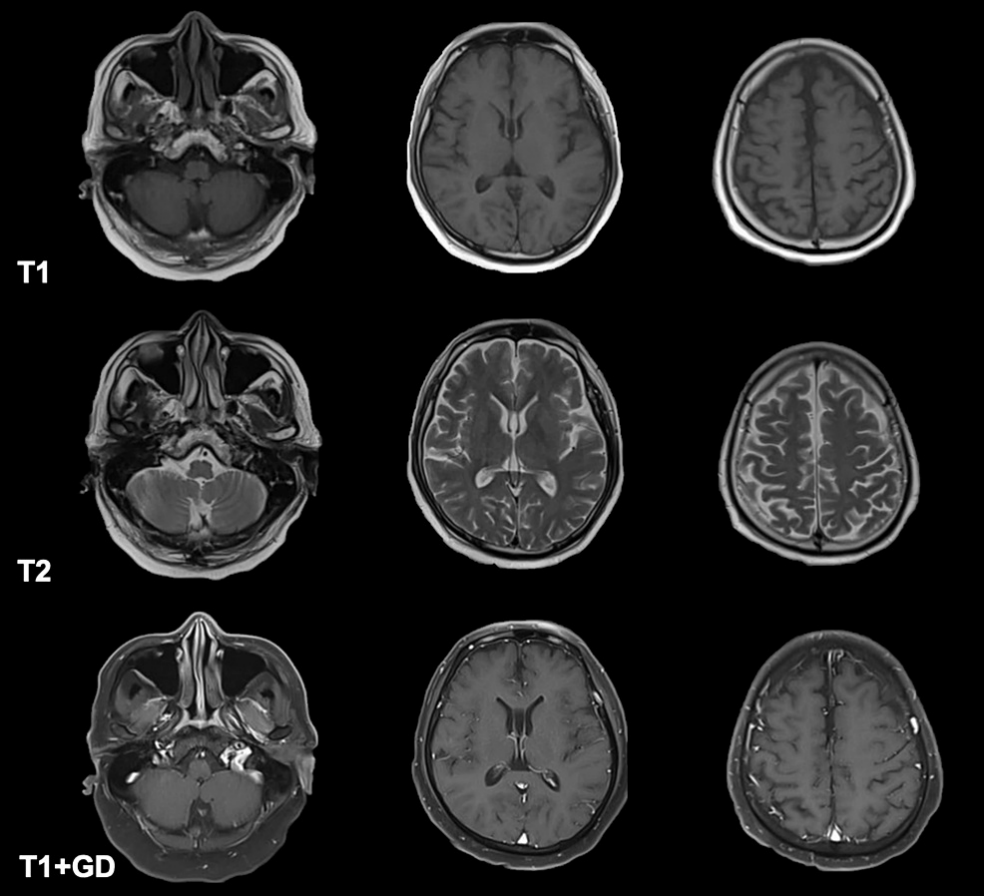
Cranial magnetic resonance imaging with contrast results Top row: axial T1 representative images, middle row: axial T2 representative images, bottom row: axial T1 with contrast representative images

Laboratory examinations (Table 1) showed hyperglycemia, mild hyponatremia, hyperkalemia, elevated inflammatory markers, normal hemoglobin and hematocrit, normal white blood cell count and platelet count, normal kidney and thyroid function, normal bilirubin and liver enzymes, normal bleeding parameters, ketonuria, normal sinus rhythm on electrocardiogram, trace serum ketones, normal acid base status on arterial blood gas, positive SARS-CoV2 reverse transcriptase polymerase chain reaction test, and bilateral middle to lower lung pneumonia on chest radiograph. No available serum nor urine assay to measure ivermectin was available. Cranial magnetic resonance imaging with contrast showed unremarkable findings (Figure 1). A two-hour video electroencephalogram showed mild to moderate diffuse cerebral dysfunction and absence of seizure activity. The patient was admitted to the progressive care unit, maintained on a high-flow nasal cannula and nasogastric tube, and was administered ceftriaxone 2 grams and azithromycin 500mg intravenously. He was then given two doses of activated charcoal (total of 100mg) and laxatives. A cerebrospinal fluid (CSF) analysis and immunological screening were no longer done since his neurologic status had significantly improved within 24 hours after initiation of treatment, where he was now noted to be awake, alert, oriented, able to follow commands, with no other focal neurologic deficits. Given the absence of abnormal findings on electroencephalogram and rapid improvement of the patient's neurologic status after administration of activated charcoal, he was then subsequently managed as a case of multifactorial encephalopathy secondary to COVID-19 infection and ivermectin toxicity. By the second hospital day, the patient's neurological symptoms had completely resolved, antibiotics were continued, and he was gradually weaned off oxygen support. He was then discharged stable after eight hospital days. The clinical outcome on the follow-up of the patient shows no residual symptoms and neurologic deficits, and he was able to resume his normal daily activities thereafter.

**Table 1:** Serum tests performed during the course of the hospital admission

Test	Results	Reference value
Hemoglobin	15.7 g/dL	13.0-17.0 g/dL
Hematocrit	44.8%	40.0-52.0
White blood cell count	5,880 mm^3^	4,800-10,800 mm^3`^
Neutrophils	83%	40-74%
Lymphocytes	9%	19-48%
Monocytes	8%	3-9%
Platelet count	347,000/mm^3^	150,000-400,000/mm^3^
Mean corpuscular volume	84 fL	82-98 fL
Mean corpuscular hematocrit	30 fL	28-33 fL
Mean corpuscular hemoglobin concentration	35%	32-38%
Erythrocyte sedimentation rate	66 mm/hour	0-20 mm/hour
C-reactive protein	2.02 mg/dL	0-0.8 mg/dL
D-dimer	638 ng/mL	0-246 ng/mL
Ferritin	1,067 ng/mL	21.81-274.6 ng/mL
Lactate dehydrogenase	225 U/L	120-246 U/L
Vitamin D	29.62 ng/mL	30.00-80.00
Serum capillary blood glucose	342 mg/dL	< 200mg/dL
Creatinine	0.97 mg/dL	0.7-1.3 mg/dL
Blood urea nitrogen	28 mg/dL	9-23 mg/dL
Serum osmolality	295 mOsm/kg	275-295 mOsm/kg
Sodium	134 mmol/L	136-145 mmol/L
Potassium	5.3 mmol/L	3.5-5.1 mmol/L
Bicarbonate	25 mmol/L	20-31 mmol/L
Thyroid stimulating hormone	1.12 uIU/mL	0.55-4.78 uIU/mL
Free T3	2.41 ng/dL	2.3-4.2 ng/dL
Free T4	1.73 ng/dL	0.89-1.76 ng/dL
Alanine transferase	41 U/L	10-49 U/L
Aspartate transferase	21 U/L	0-34 U/L
Total bilirubin	1.06 mg/dL	0.3-1.2 mg/dL
Alkaline phosphatase	91 U/L	46-116 U/L
Lipase	44 U/L	12-53 U/L
Albumin	4.32 g/dL	3.4-5.0 g/dL
Prothrombin time (control)	11.6 seconds	11.9-14.2 seconds
Prothrombin time (test)	12.5 seconds	11.9-14.2 seconds
Internationalized normalized ratio	1.06	0.9-1.19
Partial thromboplastin time	34.0 seconds	29.5-39.9 seconds
Serum ketones	15 mg/dL	Not applicable
Creatine kinase - MM	381 U/L	35-232 U/L
Arterial blood gas pH	7.40	7.35-7.45
Arterial blood gas pCO2	40.6 mmHg	35-45 mmHg
Arterial blood gas pO2	303 mmHg	80-100 mmHg
Arterial blood gas HCO3	24.7 mmol/L	22-26 mmol/L
Arterial blood gas SpO2	100.3%	95-100%
Blood cultures (2 peripheral sites)	No growth after 5 days	Negative

## Discussion

Since the start of the COVID-19 pandemic, the use of ivermectin in the prevention and treatment of SARS-CoV2 infection has gained widespread popularity. In vitro studies suggest that ivermectin acts by inhibiting the host importin alpha/beta-1 nuclear transport proteins, which are part of an intracellular transport process that viruses hijack to enhance infection by suppressing the host's antiviral response, underlying its use against viruses such as dengue virus, yellow fever virus, and Zika virus. Studies have also suggested anti-inflammatory properties, which have been hypothesized to be beneficial in SARS-CoV2 infections [3, 4, 5, 6]. Despite these studies, no clinical trials have reported a clinical benefit of ivermectin against all these viral infections. Additionally, the dose required to achieve the adequate plasma concentrations necessary for antiviral efficacy would require those up to 100-fold higher than the dose approved for use in humans [4, 7].

At therapeutic doses, ivermectin does not readily penetrate the blood-brain barrier, where GABA functions as a neurotransmitter. The therapeutic dosage recommended is 0.2-0.3mg/kg/day, with a maximum daily dosage of 0.4mg/kg/day [3, 4]. Ivermectin has a high margin of safety in humans and does not readily cause serious adverse drug reactions since it is transported out of the central nervous system by ATP-binding cassette subfamily B member 1 (ABCB1) transporter [3]. It has also been considered to be free of the potential to cause neurological adverse drug reactions due to the low drug levels in the central nervous system, except in situations of drug overdose [1, 2, 5]. Neurological adverse effects have been reported in those treated for parasitic diseases in doses above 2mg/kg/day [8, 9]. Symptoms of neurotoxicity include lethargy, drooling, tremors/seizures, gait imbalance, decrease in sensorium, and disorientation [4]. In patients with mutations of the ABCB1 transporter, intake of therapeutic doses of ivermectin will result in serious neurological symptoms such as coma, ataxia, pyramidal signs, and binocular diplopia [3, 7]. Non-neurological adverse events included pruritus, myalgia, cough, difficulty of breathing, nausea and vomiting, diarrhea, postural hypotension and serious skin reactions, and edematous swelling [8, 9].

At present, there are no local case reports in the Philippines on the serious adverse drug reactions of ivermectin in patients with SARS-CoV2 infection. There are also no guidelines that exist for the treatment of ivermectin toxicity; there is no specific antidote for ivermectin overdose and management remains to be supportive in cases of overdose. The benefit of administering activated charcoal in alleviating the adverse drug effects of ivermectin overdose is probably due to the accelerated fecal route of drug elimination, as was seen in our patient's case [1].

Our study has several limitations. There are confounding factors, such as the presence of metabolic conditions and SARS-CoV2 infection, the unavailability of a diagnostic examination to measure ivermectin levels, and the exclusion of CSF analysis as part of the diagnostic workup. In the context of the pandemic and the continued use of ivermectin for the prophylaxis and treatment of SARS-CoV2 infection, it is therefore difficult to conclude that our patient's neurological symptoms are solely associated with ivermectin toxicity as many patients who develop severe SARS-CoV2 infection will develop similar symptoms [6]. The American Association of Poison Control Center has reported similar adverse drug reactions from the use of ivermectin, for which most of the cases reported had minimal clinical effects, and its use was intended for prophylaxis or treatment of SARS-CoV2 infection. Locally, the University of the Philippines National Poison Management and Control Center has reported a total of 16 cases of ivermectin exposures, all of which had consumed the recommended daily dose of ivermectin. Most of the reported cases were unintentional, with none reporting neurological symptoms similar to our index case [10]. It is, therefore, essential for clinicians to properly educate patients on the use of ivermectin and that it has unproven benefits in the prophylaxis and treatment of SARS-CoV2 infection.

## Conclusions

In summary, we present the case of a 52-year-old Filipino male who was treated as a case of multifactorial encephalopathy secondary to ivermectin toxicity and SARS-CoV2 infection in the Philippines. Any serious non-neurologic and neurologic adverse effects must be reported to educate the public on the risks of unintentional and intentional use of ivermectin. Despite the lack of treatment guidelines for cases of Ivermectin overdose, activated charcoal was shown to be efficacious in our patient's case and should be considered as a therapeutic option. While the clinical syndrome of ivermectin toxicity has yet to be fully identified, further studies are needed to identify all the possible short-term and long-term complications with the use of ivermectin. Therefore, it is important for clinicians to recognize the adverse drug effects associated with the use of ivermectin. Lastly, it is important to highlight ivermectin has uncertain health benefits in the prevention and treatment of SARS-CoV2 infection.
